# 2-[*N*-(2,4-Difluoro­phen­yl)carbamo­yl]-3,4,5,6-tetra­fluoro­benzoic acid

**DOI:** 10.1107/S1600536809038306

**Published:** 2009-10-03

**Authors:** Duoli Guo, Gary S. Nichol, James P. Cain, Samuel H. Yalkowsky

**Affiliations:** a College of Pharmacy, The University of Arizona, 1295 N. Martin Avenue, Tucson, AZ 85721, USA; b Department of Chemistry and Biochemistry, The University of Arizona, 1306 E. University Boulevard, Tucson, AZ 85721, USA

## Abstract

The title compound, C_14_H_5_F_6_NO_3_, was synthesized by condensation of tetra­fluoro­phthalic anhydride and 2,4-difluoro­aniline. It was then recrystallized from hexane to give a nonmerohedral twin with two crystallographically unique mol­ecules in the asymmetric unit. The refined twin fraction is 0.460 (3). Torsional differences between the aryl rings and the central amide group account for the presence of two unique mol­ecules. The compound packs as double tapes formed by O—H⋯O and N—H⋯O hydrogen-bonding inter­actions between each unique mol­ecule and its symmetry equivalents.

## Related literature

For the synthesis of a related structure, see: Collin *et al.* (2001 ▶). For anti­tumor effects of thalidomide analogs, see: Ng *et al.* (2004 ▶).
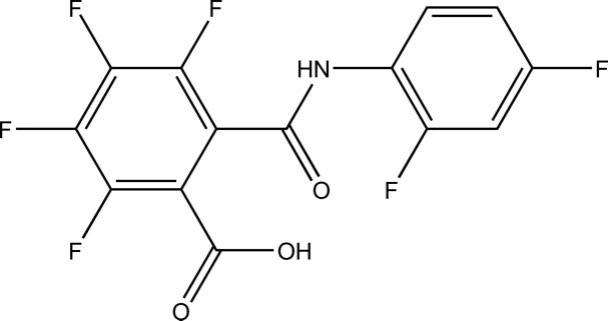


## Experimental

### 

#### Crystal data

C_14_H_5_F_6_NO_3_
*M* *_r_* = 349.19
Triclinic, 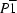

*a* = 7.5293 (4) Å
*b* = 7.6795 (5) Å
*c* = 24.1969 (15) Å
α = 89.809 (5)°
β = 82.747 (4)°
γ = 68.712 (4)°
*V* = 1291.83 (13) Å^3^
*Z* = 4
Cu *K*α radiation
μ = 1.65 mm^−1^
*T* = 100 K
0.27 × 0.19 × 0.09 mm

#### Data collection

Bruker Kappa APEXII DUO CCD diffractometer
Absorption correction: multi-scan (*TWINABS*; Sheldrick, 1996 ▶) *T*_min_ = 0.664, *T*_max_ = 0.868
7781 measured reflections
3296 independent reflections
2467 reflections with *I* > 2σ(*I*)
*R*_int_ = 0.074
θ_max_ = 58.5°

#### Refinement

*R*[*F* ^2^ > 2σ(*F* ^2^)] = 0.067
*wR*(*F* ^2^) = 0.182
*S* = 0.99
3296 reflections
434 parameters
H-atom parameters constrained
Δρ_max_ = 0.40 e Å^−3^
Δρ_min_ = −0.43 e Å^−3^

### 

Data collection: *APEX2* (Bruker, 2007 ▶); cell refinement: *SAINT* (Bruker, 2007 ▶); data reduction: *SAINT*; program(s) used to solve structure: *SHELXTL* (Sheldrick, 2008 ▶); program(s) used to refine structure: *SHELXTL*; molecular graphics: *ORTEP-3 for Windows* (Farrugia, 1997 ▶) and *Mercury* (Macrae *et al.*, 2008 ▶); software used to prepare material for publication: *SHELXTL* and local programs.

## Supplementary Material

Crystal structure: contains datablocks I, global. DOI: 10.1107/S1600536809038306/bt5063sup1.cif

Structure factors: contains datablocks I. DOI: 10.1107/S1600536809038306/bt5063Isup2.hkl

Additional supplementary materials:  crystallographic information; 3D view; checkCIF report

## Figures and Tables

**Table 1:** Hydrogen-bond geometry (Å, °)

*D*—H⋯*A*	*D*—H	H⋯*A*	*D*⋯*A*	*D*—H⋯*A*
O3—H3⋯O1^i^	0.84	1.84	2.666 (6)	168
N1—H1⋯O2^ii^	0.88	2.09	2.902 (7)	154
O53—H53⋯O51^iii^	0.84	1.84	2.675 (6)	170
N51—H51⋯O52^iv^	0.88	2.07	2.902 (8)	157

Symmetry codes: (i) 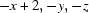; (ii) 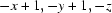; (iii) 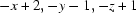; (iv) 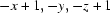.

**Fig. 1.**
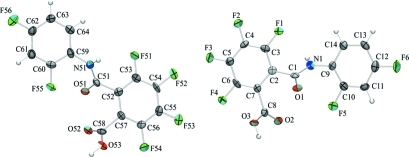
The asymmetric unit of (I) with displacment ellipsoids at the 50% probability level.

**Fig. 2.**
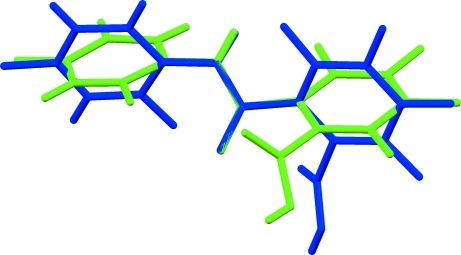
An overlay of the amide groups of molecules A (green) and B (blue) with an r.m.s. deviation of 0.0149 Å.

**Fig. 3.**
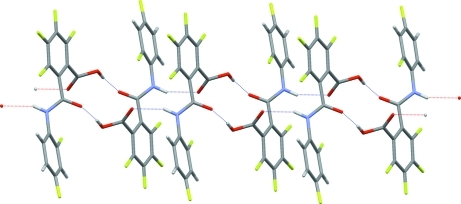
Hydrogen bonding interactions (blue dotted lines; red dotted lines indicate hydrogen bonding continuation) in (I).

**Fig. 4.**
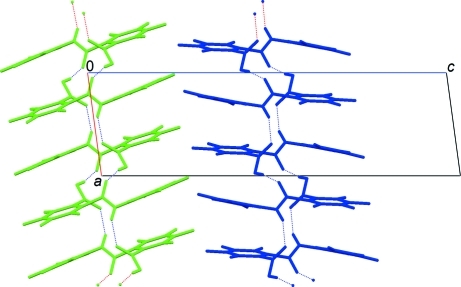
A b-axis projection of the crystal packing in (I). The colour scheme is that used in Figure 2.

## supplementary crystallographic information

### Comment

Analogs of thalidomide have demonstrated promising anti-cancer activity (Ng *et al.*, 2004). Inhibition of the angiogenesis activity of 2-(2,4-difluorophenyl)-4,5,6,7-tetrafluoroisoindoline-1,3-dione led us to synthesize and test the activity of its degradation product, 2-(2,4-difluorophenylcarbamoyl)-3,4,5,6-tetrafluorobenzoic acid, (I). In the course of doing so we determined its crystal structure.

Compound (I) was recrystallized from hexane to yield, concomitantly, small needle and prism-like crystals. Both crystal morphologies have the same unit cell parameters and data were collected on a prism crystal. Although the crystal was reasonably large the diffraction was phenomenally weak. With 60 second exposures using Cu radiation data could only be observed up to a resolution of 1 Å; the dataset was truncated at this resolution. The diffraction pattern is a non-merohedral twin with a refined twin fraction ration of 0.460 (3). A room temperature redetermination of the unit cell, using a fresh crystal, showed that twinning was still present and does not result from flash-cooling to 100 K.

The structure features two crystallographically independent molecules in the asymmetric unit. The structural discussion is limited to molecule A (atoms F1 > C14) with equivalent results for molecule B (F51 > C14) presented in square brackets. The asymmetric unit is shown in Figure 1. Figure 2 shows an overlay of both molecules,formed by overlaying the central amide group.Molecular dimensions are unexceptional. The plane of the more highly-substituted aryl ring is rotated by 69.41 (5)° [69.64 (4)°] from the amide group, and the carboxylic acid group is rotated by 58.70 (2)° [61.10 (3)°] from the aryl ring. The plane of the second aryl ring is rotated by 70.25 (4)° [66.54 (4)°] from the amide group. As can be see from Figure 3 and from the direction of rotation is different for each molecule, most easily appreciated by considering the torsion angle N1–C1–C2–C3 - 49.0 (8)° [48.5 (10)°] (similar magnitude, opposite direction).

As shown in Figures 3 and 4 the compound packs as a double tape structure with each crystallographically unique molecule hydrogen-bonded to its symmetry equivalents *via* O–H···O and N–H···O hydrogen bonding interactions. Each tape runs parallel to the *a* axis.

### Experimental

Compound (I) was synthesized by coupling tetraflurophthalic anhydride (1 g, 4.54 mmol) and 2,4-difluoroaniline (0.59 g, 4.54 mmol) using 4-dimethylaminopyridine (56 mg, 0.454 mmol) as the catalyst and dry dichloromethane (10 ml) as the solvent. The mixture was stirred at ambient temperature for 72 h. After evaporation of the solvent, the reaction mixture was partitioned between ethyl acetate and 2 N HCl. The aqueous phase was then extracted twice more with ethyl acetate. The combined extracts were evaporated to a solid *in vacuo*. The solid was treated with diethyl ether at room temperature and the undissolved residue was discarded. The remaining solution was evaporated to give a white powder, which was recrystallized from hexane.

### Refinement

All H-atoms were placed geometrically and refined with *U*_iso_(H) = 1.2Ueq(*X*) (*X* = C, H, O) and fixed distances of: O–H, 0.84 Å; N–H, 0.88 Å; C–H, 0.95 Å. The structure was refined as a non-merohedral twin (twin law: -1 0 0 / 0 -1 0 / -1 0.31 1). The fraction of the minor twin domain was 0.460 (3).

### Crystal data

**Table:**

C_14_H_5_F_6_NO_3_	*Z* = 4
*M**_r_* = 349.19	*F*(000) = 696
Triclinic, *P*1	*D*_x_ = 1.795 Mg m^−^^3^
Hall symbol: -P 1	Cu *K*α radiation, λ = 1.54178 Å
*a* = 7.5293 (4) Å	Cell parameters from 617 reflections
*b* = 7.6795 (5) Å	θ = 3.7–56.9°
*c* = 24.1969 (15) Å	µ = 1.65 mm^−^^1^
α = 89.809 (5)°	*T* = 100 K
β = 82.747 (4)°	Prism, colorless
γ = 68.712 (4)°	0.27 × 0.19 × 0.09 mm
*V* = 1291.83 (13) Å^3^	

### Data collection

**Table:**

Bruker Kappa APEXII DUO CCD diffractometer	3296 independent reflections
Radiation source: microsource	2467 reflections with *I* > 2σ(*I*)
silicon	*R*_int_ = 0.074
φ and ω scans	θ_max_ = 58.5°, θ_min_ = 1.8°
Absorption correction: multi-scan (TWINABS; Sheldrick, 1996)	*h* = −8→8
*T*_min_ = 0.664, *T*_max_ = 0.868	*k* = −8→8
7781 measured reflections	*l* = 0→26

### Refinement

**Table:**

Refinement on *F*^2^	Primary atom site location: structure-invariant direct methods
Least-squares matrix: full	Secondary atom site location: difference Fourier map
*R*[*F*^2^ > 2σ(*F*^2^)] = 0.067	Hydrogen site location: inferred from neighbouring sites
*wR*(*F*^2^) = 0.182	H-atom parameters constrained
*S* = 0.99	*w* = 1/[σ^2^(*F*_o_^2^) + (0.1222*P*)^2^] where *P* = (*F*_o_^2^ + 2*F*_c_^2^)/3
3296 reflections	(Δ/σ)_max_ < 0.001
434 parameters	Δρ_max_ = 0.40 e Å^−^^3^
0 restraints	Δρ_min_ = −0.43 e Å^−^^3^

### Special details

**Table:**

Geometry. All e.s.d.'s (except the e.s.d. in the dihedral angle between two l.s. planes) are estimated using the full covariance matrix. The cell e.s.d.'s are taken into account individually in the estimation of e.s.d.'s in distances, angles and torsion angles; correlations between e.s.d.'s in cell parameters are only used when they are defined by crystal symmetry. An approximate (isotropic) treatment of cell e.s.d.'s is used for estimating e.s.d.'s involving l.s. planes.
Refinement. Refinement of *F*^2^ against ALL reflections. The weighted *R*-factor *wR* and goodness of fit *S* are based on *F*^2^, conventional *R*-factors *R* are based on *F*, with *F* set to zero for negative *F*^2^. The threshold expression of *F*^2^ > σ(*F*^2^) is used only for calculating *R*-factors(gt) *etc*. and is not relevant to the choice of reflections for refinement. *R*-factors based on *F*^2^ are statistically about twice as large as those based on *F*, and *R*- factors based on ALL data will be even larger.

### Fractional atomic coordinates and isotropic or equivalent isotropic displacement parameters (Å^2^)

**Table:**

	*x*	*y*	*z*	*U*_iso_*/*U*_eq_	
F1	0.6312 (6)	0.7940 (5)	0.07802 (17)	0.0314 (10)	
F2	0.5202 (6)	0.7903 (6)	0.18695 (17)	0.0342 (11)	
F3	0.5293 (7)	0.4660 (6)	0.23442 (17)	0.0380 (12)	
F4	0.6469 (7)	0.1469 (6)	0.17134 (17)	0.0368 (11)	
F5	0.8026 (6)	0.3185 (6)	−0.11126 (18)	0.0310 (10)	
F6	0.9832 (7)	0.7035 (7)	−0.23925 (18)	0.0441 (13)	
O1	0.9457 (6)	0.3317 (6)	−0.0108 (2)	0.0233 (11)	
O2	0.6865 (7)	0.1123 (7)	0.01592 (19)	0.0252 (12)	
O3	0.9119 (7)	−0.0116 (6)	0.0722 (2)	0.0279 (12)	
H3	0.9411	−0.1078	0.0513	0.034*	
N1	0.6934 (8)	0.5969 (8)	−0.0258 (2)	0.0208 (13)	
H1	0.5782	0.6737	−0.0115	0.025*	
C1	0.7927 (10)	0.4594 (10)	0.0057 (3)	0.0221 (17)	
C2	0.7033 (10)	0.4683 (10)	0.0659 (3)	0.0233 (17)	
C3	0.6390 (10)	0.6307 (10)	0.0997 (3)	0.0252 (17)	
C4	0.5817 (10)	0.6302 (10)	0.1557 (3)	0.0257 (17)	
C5	0.5853 (11)	0.4681 (11)	0.1803 (3)	0.0270 (18)	
C6	0.6523 (11)	0.3034 (10)	0.1465 (3)	0.0289 (19)	
C7	0.7083 (10)	0.3010 (10)	0.0908 (3)	0.0210 (16)	
C8	0.7685 (10)	0.1238 (10)	0.0551 (3)	0.0229 (16)	
C9	0.7661 (10)	0.6233 (10)	−0.0807 (3)	0.0216 (16)	
C10	0.8250 (11)	0.4836 (10)	−0.1232 (3)	0.0285 (18)	
C11	0.8983 (11)	0.5061 (10)	−0.1755 (3)	0.0288 (18)	
H11	0.9396	0.4074	−0.2032	0.035*	
C12	0.9107 (11)	0.6748 (12)	−0.1869 (3)	0.032 (2)	
C13	0.8517 (11)	0.8235 (11)	−0.1472 (3)	0.0289 (18)	
H13	0.8582	0.9414	−0.1566	0.035*	
C14	0.7839 (11)	0.7933 (11)	−0.0938 (3)	0.0285 (18)	
H14	0.7487	0.8899	−0.0655	0.034*	
F51	0.7042 (6)	0.2634 (6)	0.41888 (17)	0.0305 (10)	
F52	0.6881 (7)	0.2199 (6)	0.31022 (18)	0.0402 (12)	
F53	0.7306 (6)	−0.1187 (6)	0.26531 (18)	0.0383 (11)	
F54	0.7893 (7)	−0.4145 (6)	0.33004 (18)	0.0350 (11)	
F55	0.6962 (6)	−0.1608 (5)	0.60603 (17)	0.0311 (11)	
F56	0.8200 (6)	0.2169 (6)	0.73646 (18)	0.0413 (12)	
O51	0.9354 (7)	−0.1677 (7)	0.5081 (2)	0.0264 (12)	
O52	0.6925 (7)	−0.3957 (7)	0.4849 (2)	0.0259 (12)	
O53	0.9737 (7)	−0.5342 (6)	0.4286 (2)	0.0279 (12)	
H53	0.9875	−0.6255	0.4491	0.034*	
N51	0.6738 (9)	0.1005 (8)	0.5249 (2)	0.0251 (14)	
H51	0.5713	0.1778	0.5119	0.030*	
C51	0.7952 (11)	−0.0447 (10)	0.4919 (3)	0.0245 (17)	
C52	0.7609 (10)	−0.0563 (10)	0.4324 (3)	0.0253 (17)	
C53	0.7284 (11)	0.0938 (10)	0.3988 (3)	0.0269 (17)	
C54	0.7213 (11)	0.0727 (10)	0.3424 (3)	0.0270 (18)	
C55	0.7409 (11)	−0.1006 (11)	0.3196 (3)	0.0268 (18)	
C56	0.7748 (10)	−0.2496 (10)	0.3531 (3)	0.0236 (17)	
C57	0.7811 (10)	−0.2303 (10)	0.4091 (3)	0.0221 (17)	
C58	0.8084 (11)	−0.3979 (10)	0.4460 (3)	0.0246 (17)	
C59	0.7037 (11)	0.1344 (10)	0.5794 (3)	0.0272 (18)	
C60	0.7205 (10)	0.0006 (10)	0.6193 (3)	0.0252 (17)	
C61	0.7593 (11)	0.0240 (11)	0.6723 (3)	0.0307 (18)	
H61	0.7755	−0.0707	0.6986	0.037*	
C62	0.7733 (12)	0.1938 (11)	0.6849 (3)	0.032 (2)	
C63	0.7495 (11)	0.3315 (11)	0.6487 (3)	0.0299 (19)	
H63	0.7575	0.4470	0.6595	0.036*	
C64	0.7138 (11)	0.3034 (11)	0.5961 (3)	0.0300 (18)	
H64	0.6955	0.4007	0.5706	0.036*	

### Atomic displacement parameters (Å^2^)

**Table:**

	*U*^11^	*U*^22^	*U*^33^	*U*^12^	*U*^13^	*U*^23^
F1	0.040 (3)	0.024 (2)	0.032 (2)	−0.014 (2)	−0.003 (2)	−0.0027 (19)
F2	0.040 (3)	0.029 (2)	0.032 (2)	−0.010 (2)	−0.005 (2)	−0.006 (2)
F3	0.047 (3)	0.033 (3)	0.022 (2)	−0.002 (2)	−0.001 (2)	0.0010 (19)
F4	0.052 (3)	0.021 (2)	0.032 (2)	−0.008 (2)	−0.002 (2)	0.0045 (19)
F5	0.029 (2)	0.027 (2)	0.038 (3)	−0.010 (2)	−0.013 (2)	0.0016 (19)
F6	0.052 (3)	0.051 (3)	0.023 (2)	−0.016 (2)	0.006 (2)	0.007 (2)
O1	0.015 (3)	0.018 (3)	0.034 (3)	−0.002 (2)	−0.005 (2)	−0.006 (2)
O2	0.024 (3)	0.027 (3)	0.028 (3)	−0.013 (2)	−0.003 (2)	−0.003 (2)
O3	0.035 (3)	0.014 (3)	0.033 (3)	−0.004 (2)	−0.015 (2)	−0.001 (2)
N1	0.005 (3)	0.024 (3)	0.031 (3)	−0.001 (2)	−0.009 (3)	0.005 (3)
C1	0.027 (4)	0.019 (4)	0.030 (4)	−0.016 (4)	−0.011 (3)	−0.001 (3)
C2	0.019 (4)	0.033 (4)	0.029 (4)	−0.020 (3)	−0.009 (3)	0.001 (3)
C3	0.016 (4)	0.018 (4)	0.038 (4)	−0.003 (3)	−0.005 (4)	0.001 (3)
C4	0.027 (4)	0.029 (4)	0.025 (4)	−0.013 (3)	−0.007 (3)	−0.007 (3)
C5	0.024 (4)	0.025 (4)	0.029 (4)	−0.004 (3)	−0.009 (3)	0.000 (3)
C6	0.030 (4)	0.022 (4)	0.036 (5)	−0.009 (4)	−0.009 (4)	0.015 (4)
C7	0.016 (4)	0.024 (4)	0.024 (4)	−0.008 (3)	−0.006 (3)	0.004 (3)
C8	0.023 (4)	0.028 (4)	0.021 (4)	−0.014 (3)	−0.001 (3)	0.002 (3)
C9	0.015 (4)	0.026 (4)	0.022 (4)	−0.004 (3)	−0.008 (3)	0.002 (3)
C10	0.031 (4)	0.026 (4)	0.031 (4)	−0.012 (4)	−0.005 (4)	0.005 (3)
C11	0.031 (4)	0.018 (4)	0.031 (4)	−0.002 (3)	−0.004 (4)	−0.005 (3)
C12	0.024 (4)	0.044 (5)	0.025 (4)	−0.009 (4)	−0.003 (3)	0.003 (4)
C13	0.027 (4)	0.024 (4)	0.038 (5)	−0.009 (3)	−0.011 (4)	0.012 (4)
C14	0.032 (4)	0.032 (4)	0.028 (4)	−0.015 (4)	−0.014 (3)	0.006 (4)
F51	0.035 (2)	0.022 (2)	0.036 (2)	−0.0108 (19)	−0.010 (2)	0.0055 (19)
F52	0.052 (3)	0.035 (3)	0.034 (3)	−0.015 (2)	−0.011 (2)	0.013 (2)
F53	0.047 (3)	0.042 (3)	0.027 (2)	−0.017 (2)	−0.007 (2)	0.005 (2)
F54	0.042 (3)	0.026 (2)	0.035 (3)	−0.007 (2)	−0.011 (2)	−0.005 (2)
F55	0.036 (2)	0.018 (2)	0.039 (3)	−0.010 (2)	0.000 (2)	0.0043 (19)
F56	0.036 (3)	0.051 (3)	0.036 (3)	−0.015 (2)	−0.007 (2)	−0.008 (2)
O51	0.031 (3)	0.019 (3)	0.031 (3)	−0.011 (3)	−0.006 (2)	0.001 (2)
O52	0.024 (3)	0.021 (3)	0.033 (3)	−0.008 (2)	−0.001 (3)	0.005 (2)
O53	0.023 (3)	0.020 (3)	0.041 (3)	−0.008 (2)	−0.004 (2)	0.009 (2)
N51	0.024 (3)	0.022 (3)	0.027 (3)	−0.005 (3)	−0.009 (3)	0.002 (3)
C51	0.029 (4)	0.024 (4)	0.028 (4)	−0.016 (4)	−0.009 (4)	0.010 (3)
C52	0.019 (4)	0.026 (4)	0.030 (4)	−0.006 (3)	−0.005 (3)	−0.002 (3)
C53	0.021 (4)	0.024 (4)	0.036 (5)	−0.008 (3)	−0.005 (4)	0.005 (4)
C54	0.022 (4)	0.024 (4)	0.033 (4)	−0.007 (3)	−0.005 (3)	0.018 (4)
C55	0.018 (4)	0.033 (5)	0.025 (4)	−0.002 (3)	−0.006 (3)	0.003 (4)
C56	0.018 (4)	0.026 (4)	0.032 (4)	−0.013 (3)	−0.007 (3)	−0.001 (4)
C57	0.014 (4)	0.022 (4)	0.028 (4)	−0.005 (3)	−0.003 (3)	0.006 (3)
C58	0.021 (4)	0.014 (4)	0.038 (5)	−0.003 (3)	−0.011 (4)	−0.001 (3)
C59	0.024 (4)	0.027 (4)	0.026 (4)	−0.004 (3)	−0.003 (3)	0.008 (3)
C60	0.015 (4)	0.018 (4)	0.041 (5)	−0.004 (3)	−0.005 (3)	−0.001 (3)
C61	0.031 (4)	0.028 (4)	0.028 (4)	−0.005 (3)	−0.004 (3)	0.003 (3)
C62	0.033 (5)	0.029 (5)	0.025 (4)	−0.001 (4)	−0.003 (4)	−0.003 (4)
C63	0.032 (5)	0.026 (4)	0.033 (4)	−0.011 (4)	−0.005 (4)	−0.003 (4)
C64	0.029 (4)	0.025 (4)	0.036 (5)	−0.010 (4)	−0.004 (4)	0.006 (4)

### Geometric parameters (Å, °)

**Table:**

F1—C3	1.342 (8)	F51—C53	1.332 (8)
F2—C4	1.342 (8)	F52—C54	1.336 (8)
F3—C5	1.326 (8)	F53—C55	1.337 (9)
F4—C6	1.353 (8)	F54—C56	1.348 (8)
F5—C10	1.364 (8)	F55—C60	1.361 (8)
F6—C12	1.365 (9)	F56—C62	1.367 (9)
O1—C1	1.230 (9)	O51—C51	1.240 (8)
O2—C8	1.214 (8)	O52—C58	1.195 (9)
O3—H3	0.840	O53—H53	0.840
O3—C8	1.309 (8)	O53—C58	1.321 (9)
N1—H1	0.880	N51—H51	0.880
N1—C1	1.349 (9)	N51—C51	1.341 (10)
N1—C9	1.416 (9)	N51—C59	1.405 (10)
C1—C2	1.517 (10)	C51—C52	1.504 (10)
C2—C3	1.388 (10)	C52—C53	1.375 (11)
C2—C7	1.408 (10)	C52—C57	1.401 (10)
C3—C4	1.369 (10)	C53—C54	1.384 (11)
C4—C5	1.371 (11)	C54—C55	1.393 (11)
C5—C6	1.400 (11)	C55—C56	1.366 (11)
C6—C7	1.359 (11)	C56—C57	1.371 (11)
C7—C8	1.506 (10)	C57—C58	1.532 (10)
C9—C10	1.396 (11)	C59—C60	1.390 (10)
C9—C14	1.391 (10)	C59—C64	1.391 (11)
C10—C11	1.351 (10)	C60—C61	1.378 (11)
C11—H11	0.950	C61—H61	0.950
C11—C12	1.357 (11)	C61—C62	1.383 (11)
C12—C13	1.399 (11)	C62—C63	1.348 (11)
C13—H13	0.950	C63—H63	0.950
C13—C14	1.378 (11)	C63—C64	1.366 (11)
C14—H14	0.950	C64—H64	0.950
			
H3—O3—C8	109.5	H53—O53—C58	109.5
H1—N1—C1	118.5	H51—N51—C51	118.6
H1—N1—C9	118.5	H51—N51—C59	118.6
C1—N1—C9	123.0 (6)	C51—N51—C59	122.7 (6)
O1—C1—N1	125.2 (7)	O51—C51—N51	122.9 (6)
O1—C1—C2	119.4 (6)	O51—C51—C52	118.8 (7)
N1—C1—C2	115.4 (6)	N51—C51—C52	118.2 (6)
C1—C2—C3	122.6 (6)	C51—C52—C53	122.1 (7)
C1—C2—C7	118.8 (6)	C51—C52—C57	118.6 (6)
C3—C2—C7	118.1 (7)	C53—C52—C57	119.0 (7)
F1—C3—C2	120.4 (7)	F51—C53—C52	121.6 (6)
F1—C3—C4	118.0 (6)	F51—C53—C54	117.7 (7)
C2—C3—C4	121.6 (7)	C52—C53—C54	120.7 (7)
F2—C4—C3	119.8 (6)	F52—C54—C53	120.1 (7)
F2—C4—C5	119.4 (6)	F52—C54—C55	119.9 (7)
C3—C4—C5	120.8 (7)	C53—C54—C55	119.9 (7)
F3—C5—C4	121.4 (7)	F53—C55—C54	119.4 (7)
F3—C5—C6	120.7 (7)	F53—C55—C56	121.4 (7)
C4—C5—C6	117.9 (7)	C54—C55—C56	119.1 (7)
F4—C6—C5	116.7 (7)	F54—C56—C55	117.6 (6)
F4—C6—C7	120.9 (7)	F54—C56—C57	120.8 (7)
C5—C6—C7	122.3 (7)	C55—C56—C57	121.5 (7)
C2—C7—C6	119.4 (7)	C52—C57—C56	119.8 (7)
C2—C7—C8	119.5 (6)	C52—C57—C58	120.1 (6)
C6—C7—C8	121.0 (6)	C56—C57—C58	120.1 (7)
O2—C8—O3	125.7 (7)	O52—C58—O53	127.9 (7)
O2—C8—C7	122.0 (6)	O52—C58—C57	122.0 (7)
O3—C8—C7	112.3 (6)	O53—C58—C57	110.1 (7)
N1—C9—C10	122.7 (6)	N51—C59—C60	120.9 (7)
N1—C9—C14	119.7 (6)	N51—C59—C64	121.8 (7)
C10—C9—C14	117.5 (7)	C60—C59—C64	117.2 (7)
F5—C10—C9	118.0 (6)	F55—C60—C59	119.2 (6)
F5—C10—C11	118.8 (6)	F55—C60—C61	117.9 (7)
C9—C10—C11	123.2 (7)	C59—C60—C61	122.9 (7)
C10—C11—H11	121.2	C60—C61—H61	122.0
C10—C11—C12	117.6 (7)	C60—C61—C62	116.0 (7)
H11—C11—C12	121.2	H61—C61—C62	122.0
F6—C12—C11	119.5 (7)	F56—C62—C61	117.0 (7)
F6—C12—C13	117.6 (7)	F56—C62—C63	119.6 (7)
C11—C12—C13	122.9 (7)	C61—C62—C63	123.4 (7)
C12—C13—H13	121.1	C62—C63—H63	120.4
C12—C13—C14	117.8 (7)	C62—C63—C64	119.3 (7)
H13—C13—C14	121.1	H63—C63—C64	120.4
C9—C14—C13	120.9 (7)	C59—C64—C63	121.0 (7)
C9—C14—H14	119.6	C59—C64—H64	119.5
C13—C14—H14	119.6	C63—C64—H64	119.5
			
C9—N1—C1—O1	−7.7 (10)	C59—N51—C51—O51	5.3 (11)
C9—N1—C1—C2	172.3 (6)	C59—N51—C51—C52	−173.6 (6)
O1—C1—C2—C3	131.0 (7)	O51—C51—C52—C53	−130.4 (8)
O1—C1—C2—C7	−40.5 (9)	O51—C51—C52—C57	43.4 (10)
N1—C1—C2—C3	−49.0 (8)	N51—C51—C52—C53	48.5 (10)
N1—C1—C2—C7	139.5 (6)	N51—C51—C52—C57	−137.7 (7)
C1—C2—C3—F1	8.5 (10)	C51—C52—C53—F51	−7.6 (11)
C1—C2—C3—C4	−171.5 (7)	C51—C52—C53—C54	171.8 (7)
C7—C2—C3—F1	−180.0 (6)	C57—C52—C53—F51	178.6 (6)
C7—C2—C3—C4	0.0 (11)	C57—C52—C53—C54	−2.0 (12)
F1—C3—C4—F2	0.0 (10)	F51—C53—C54—F52	−1.1 (11)
F1—C3—C4—C5	179.7 (6)	F51—C53—C54—C55	−178.4 (6)
C2—C3—C4—F2	−180.0 (6)	C52—C53—C54—F52	179.5 (7)
C2—C3—C4—C5	−0.3 (11)	C52—C53—C54—C55	2.2 (12)
F2—C4—C5—F3	0.0 (11)	F52—C54—C55—F53	1.6 (11)
F2—C4—C5—C6	−179.3 (6)	F52—C54—C55—C56	−180.0 (7)
C3—C4—C5—F3	−179.7 (6)	C53—C54—C55—F53	178.9 (7)
C3—C4—C5—C6	1.1 (11)	C53—C54—C55—C56	−2.7 (11)
F3—C5—C6—F4	3.4 (10)	F53—C55—C56—F54	−2.8 (11)
F3—C5—C6—C7	179.1 (7)	F53—C55—C56—C57	−178.5 (6)
C4—C5—C6—F4	−177.4 (6)	C54—C55—C56—F54	178.8 (6)
C4—C5—C6—C7	−1.6 (11)	C54—C55—C56—C57	3.1 (11)
F4—C6—C7—C2	176.9 (6)	F54—C56—C57—C52	−178.4 (6)
F4—C6—C7—C8	−0.8 (11)	F54—C56—C57—C58	1.1 (10)
C5—C6—C7—C2	1.4 (11)	C55—C56—C57—C52	−2.9 (11)
C5—C6—C7—C8	−176.3 (7)	C55—C56—C57—C58	176.6 (7)
C1—C2—C7—C6	171.3 (6)	C51—C52—C57—C56	−171.7 (6)
C1—C2—C7—C8	−11.0 (9)	C51—C52—C57—C58	8.7 (10)
C3—C2—C7—C6	−0.6 (10)	C53—C52—C57—C56	2.3 (11)
C3—C2—C7—C8	177.2 (6)	C53—C52—C57—C58	−177.2 (7)
C2—C7—C8—O2	−57.0 (9)	C52—C57—C58—O52	59.1 (10)
C2—C7—C8—O3	124.7 (7)	C52—C57—C58—O53	−119.9 (7)
C6—C7—C8—O2	120.8 (8)	C56—C57—C58—O52	−120.4 (8)
C6—C7—C8—O3	−57.6 (9)	C56—C57—C58—O53	60.5 (9)
C1—N1—C9—C10	57.0 (9)	C51—N51—C59—C60	−57.9 (10)
C1—N1—C9—C14	−121.9 (7)	C51—N51—C59—C64	123.5 (8)
N1—C9—C10—F5	3.1 (10)	N51—C59—C60—F55	−3.9 (11)
N1—C9—C10—C11	−178.6 (7)	N51—C59—C60—C61	176.7 (7)
C14—C9—C10—F5	−178.0 (6)	C64—C59—C60—F55	174.8 (6)
C14—C9—C10—C11	0.3 (11)	C64—C59—C60—C61	−4.6 (11)
F5—C10—C11—C12	177.0 (6)	F55—C60—C61—C62	−176.8 (6)
C9—C10—C11—C12	−1.3 (11)	C59—C60—C61—C62	2.6 (11)
C10—C11—C12—F6	−179.7 (7)	C60—C61—C62—F56	−177.2 (7)
C10—C11—C12—C13	0.1 (11)	C60—C61—C62—C63	0.6 (12)
F6—C12—C13—C14	−178.1 (6)	F56—C62—C63—C64	176.3 (7)
C11—C12—C13—C14	2.1 (11)	C61—C62—C63—C64	−1.5 (12)
C12—C13—C14—C9	−3.1 (10)	C62—C63—C64—C59	−0.7 (12)
N1—C9—C14—C13	−179.1 (6)	N51—C59—C64—C63	−177.7 (7)
C10—C9—C14—C13	1.9 (10)	C60—C59—C64—C63	3.6 (11)

### Hydrogen-bond geometry (Å, °)

**Table:**

*D*—H···*A*	*D*—H	H···*A*	*D*···*A*	*D*—H···*A*
O3—H3···O1^i^	0.84	1.84	2.666 (6)	168
N1—H1···O2^ii^	0.88	2.09	2.902 (7)	154
O53—H53···O51^iii^	0.84	1.84	2.675 (6)	170
N51—H51···O52^iv^	0.88	2.07	2.902 (8)	157

Symmetry codes: (i) −*x*+2, −*y*, −*z*; (ii) −*x*+1, −*y*+1, −*z*; (iii) −*x*+2, −*y*−1, −*z*+1; (iv) −*x*+1, −*y*, −*z*+1.
